# Supplementary material for: Patterns of Intron Gain and Loss in Fungi
Source: PLoS Biol. 2004 Nov 30;2(12):e422. doi: 10.1371/journal.pbio.0020422 (PMC532390; doi:10.1371/journal.pbio.0020422)
Supplement: Table S1 — Also available at http://genes.mit.edu/NielsenEtAl/. (4.3 MB ZIP). [file pbio.0020422.st001.zip › NielsenEtAl/html/159.html]

AN6211.1.NCU08298.1.MG01523.1.FG01969.1


```
 CLUSTAL W (1.82) Multiple Sequence Alignments - Introns Inserted


Sequence 1: MG01523.1	836 aa
Sequence 2: FG01969.1	821 aa
Sequence 3: NCU08298.1	841 aa
Sequence 4: AN6211.1	837 aa
Alignment Length: 901 aa
Number Identitical Residues: 300 aa
Alignment Score (without introns) 15901


MG01523.1 	MSVLLRAGARSITLTG--GVFPTECTSLGRGQRLTARSWNGYVGYAIGHRAMVTR-SPAE
NCU08298.1	-MARLGQLTFAISSTGRRSPLARSLTQSGALLRAPARTKPSLLSFQQTRHGFFASGDNKP
FG01969.1 	-MRHYRGSLRSLHRIP--SRFN-NHTRLPR-RRLTPRQHRQFW------TSHTKR-DPKE
AN6211.1  	---MGAARAFCIRFAP-----PAALIYVGGRVLWSIEAARNNS------QLFLLHSDPKA
          	          .:                      . .                  ..   

MG01523.1 	KRRIRFRRSWSSIVVGTGILTYTLYPWEDRGQARQLATDQNRRRIHPNHKDGSHEQTTKA
NCU08298.1	KPKTRVPRSLSAVLLGS-LLVFSLNPTAD--VVQTFDSTDKTAKTDRSSDTDLFAQELES
FG01969.1 	EEQQ--RSTLPVAVITGGLLIWWLYPSDD---FAQLSGKQARLRNDKDSGQDKSRDKSQN
AN6211.1  	AYSTTSTQCARGQVHHS-------------------VGEGRPGRSNTDRVEGSFHSSEP-
          	             :                             : . .   .   .    

MG01523.1 	SQS--GSGAWFNFAGSFEGLSTGLSKVADVDWGTDKVLDYIIPDYFRLVPGYLRKLQREL
NCU08298.1	HGKIDDSSTWSKLVDGFEGFSA--MTSAEAGKLSNKLVDLVLPEWSKLIPGYIRKLQREL
FG01969.1 	SESDADQSAWINFSRRFEAFST--LNSLEFSSFPDKIVNSLLPEWSRLIPGYVRKLQREL
AN6211.1  	-----DSSIWSSMMQRFEGVKQ--SVGSPEWIELDQLKNYIIPDWTKFLPETVQKLQREL
          	     ... * .:   **...             ::: : ::*:: :::*  ::******

MG01523.1 	SMSPGSLANEIWEEAHDPSTHPEIRYAAKVRVSTDLCEEEQMFREYRRMVTRVALARYLD
NCU08298.1	NMAPGSLAAEIWEEARDPAINPEIQYSAKVRVSEDLCDEEKTFLARRKKITAVALAHYLG
FG01969.1 	SMSPGSLADEIWHDAHDPLINPEIQYSATVRVSPDICDEEKEYLSRRKRVARVGLAKYLG
AN6211.1  	SMAPGSLADEIWREAHDPDLHPEILREATVRVGNSLCDDELEFRRKRKKHAVKALSAYLN
          	.*:***** ***.:*:**  :***   *.***. .:*::*  :   *:  :  .*: **.

MG01523.1 	LKEEDVNPEDVPVIGMCGSGGGLRALVAGAGSMSAAADDGLFDCVTYTAGVSGSCWLQSL
NCU08298.1	IKEEDVNPEDVPTIAICGSGGGLRALVAGTGSYMAAAEDGLFDCITYTSGVSGSCWLQSL
FG01969.1 	LKEDEVHPDDVPTIAMCGSGGGLRALIAGSGSILATEEDGLFDCVTYTSGVSGSCWLQAL
AN6211.1  	IPEEDIHPDDVPIISMCGSGGGLRALVAGTGSYLATQEAGLWDCVTYTAGVSGSCWLQVL
          	: *::::*:*** *.:**********:**:**  *: : **:**:***:********* *

MG01523.1 	YYSSLTNCSLDKLINHLKARLGVHIAFPPAAFQALVSAPTNKSLLSGLVEKLKGDPNADF
NCU08298.1	YFSSLAGNDFQRLINHLKARLGIHIAYPPTAFSALLSAPTNKLLLTAVVEKLKGDPEADF
FG01969.1 	NLTSFNQGSLKKLIEHLKARSSTHIAYPPEAFQALASMPTNKYLLSGMVEKLKGDPKADF
AN6211.1  	YHSSITGCNFTRLVAHLKNRLGVHMAFPPAALKLLTHAPTNKYLLSGLVQKLKGDPGADF
          	  :*:   .: :*: *** * . *:*:** *:. *   **** **:.:*:****** ***

MG01523.1 	GLVDVYGILLAARLLVPKGELGIDDRNYKLSNQRQYIQSGANPMPIYTAVRHEIPGVDDG
NCU08298.1	GLVDAYGLLLAARLFIPKGELGINAQDFKLSHQQEYIRYGQNPMPIYTAVRHEIPELDEA
FG01969.1 	GLVDVYGVLLAARYLVPKGDLGVNDRDFKLSNQRQYVQYGQLPLPIYTAVRHEIPNLPEA
AN6211.1  	GLVDIYGMLLAARLLVPKGELGASDFDLKLSNQARNIASGAHPLPIYTAVRHEIPILEEK
          	**** **:***** ::***:** .  : ***:* . :  *  *:*********** : : 

MG01523.1 	SPSEIPTATEEQKEQAKQEAWFQ0WFEISPYEFFCEEFEAGIPTWAMGRRFQDGKDVALP
NCU08298.1	D-NEQSHASEAAKAEAKKEAWFQ~WFEMTPFELFCEEFAAGIPTWAMGRSFQDGVNLPTT
FG01969.1 	S-NQGPIEAEIAKEEAKKEAWFQ~WYEITPYEFFCEEFGAGIPTWALGRRFKSGRDIAPE
AN6211.1  	AEKNEKPMPEDLIKESQSESWFQ~WFEFTPYEFFCEELNAGIPTWALGRHFNGGKNEVPA
          	  .:    .*    :::.*:*** *:*::*:*:****: *******:** *:.* :    

MG01523.1 	GGSHLPEIRMPLLLGMFGSAFCATLSHYYREIRPLVQNLSGWIGFDELITGRNEDLSKVH
NCU08298.1	EGFYLPEIRLPVLLGIWGSAFCATLSHYYREIRPLIQSLGGFQAVDNLIWGHNENLSKVH
FG01969.1 	HGFHLPEIRLPLLMGIFGSAFCATLSHYYREIKPLVQGLTGFGAIDDLISTRDDDLIKVH
AN6211.1  	GTAPIPELHVGSLMGVWGSAFCATLSHYYKEIRPLLRGIAGFGGIDSLIQGKSKDLIRVH
          	    :**:::  *:*::************:**:**::.: *: ..*.**  :..:* :**

MG01523.1 	PIDPTALPNPVYQMNGKLPKTTPPGVIKSEYIKLMDAGMSNNLPIYPLLRPGREVEIVIA
NCU08298.1	PIDPATIPNFIHGMNGKLPPTVPRLAYDSDYIQLMDSGMSNNLPIYPLLRPGRNVDIIVA
FG01969.1 	PIDPAKIPNFAYGMHGKLPETTPESIYDNEYIQLMDAGMSNNLPIYPLLRPGRDVDVLVA
AN6211.1  	PIDPATIPNYVLGMKDQLPPSCPESIFQSQHLRLMDAGMSNNLPIYPLLRPGRDVDIIIA
          	****: :**    *:.:** : *    ..::::***:****************:*::::*

MG01523.1 	FDASADIKTDNWLSVADGYARQRGIKGWPVGIGWPKETDPTEDTVKQLEDARANSQAEAD
NCU08298.1	FDASADIKTDNWLSVVEGYARQRGIKGWPIGAGWPKASASASTTARELKEAEEATEAEAD
FG01969.1 	FDASADIKTDNWLSVADGYARQRNIKGWPVGIGWPKPGEATSQVVEELDEAQAKSTREAE
AN6211.1  	FDNSADIKQENWLSVVDGYARQRGIKGWPIGAGWPRAADTLKETEQSLREPENISEEALN
          	** ***** :*****.:******.*****:* ***:   . . . ..* :..  :    :

MG01523.1 	QKLQLAQHDQSQSQVEQPGENKDTAKGQDGQ-VKNKYERAAESLGYCTVWVGTTEERETE
NCU08298.1	NKLAQAKADQAIRHANDPKVKPEGDSDQSLMSQQEKYDQTAHELGYCTVWVGTTEERRTD
FG01969.1 	SKLREAKKEQKELRQEAHEEGKQVMAENDKT-KFEHGDQESGDLGYCTVWVGTNQERSSK
AN6211.1  	RRVSEAQQSSTHEHAHSSSVTTKQTTTNPDF----QPSPADTDLDYCNVWVGTMQERLSD
          	 ::  *: ..   : .      .    :       : .    .*.**.***** :** :.

MG01523.1 	-PPPPLKTAEDT-----------EKWRLMEPGAGIAVVYLPFVANDKVEG----------
NCU08298.1	-DPPPP-PVDDT-----------SSWKLMEPNAGIAVVYMPFLANEKVSSS---------
FG01969.1 	-PPPPSKALSGD-----------NSWQLMEPEAGIAVVYLPFISNDKVPG----------
AN6211.1  	KEPPPSKRLFDPRQSSSDPDHRESDFHLMRPDAGIAVVYFPLLPNSSAPDLPSSTSLTKP
          	. *** .   .  .:::...  ...::**.* *******:*::.*... . .:::: :..

MG01523.1 	-------------------------------VDPATSDYMSTWNFVYTPEQVDKVVSLAK
NCU08298.1	-------------------------------VDPASSDYMSTWNFVYTPEQVDQVVALAR
FG01969.1 	-------------------------------ISPGTTDYLSTWNFIYTPEQIDNVVELAR
AN6211.1  	SPSDQHSGSENNSSAQDTIDTTKPLSPHPGTINPNVDDFLSTWNFVYTPEQIDAVVGLAK
          	:.:.. :.:...:::..: .::.. :. ..::.*   *::*****:*****:* ** **:

MG01523.1 	ANYGEGRDQIKATVRAVYERKKIMREKREEQTRREHYRRLVRLGI-A~DKLGEGDHFS--
NCU08298.1	ANYDEGKEKIRATVRAVYERKKNSRLEREKRMAEERQRRKVRLGIDV1VKVSFIDPFASG
FG01969.1 	ANYNEGKQQIRDTIRGVYERKKKLREEAEKAQREDRYRSLMRRGE-G~VRLGEGDHFS--
AN6211.1  	ANFAQGEQQVKRVVRAVYERKKKDRLQREERMHGPHKTGLVLS----~------------
          	**: :*.:::: .:*.******  * : *:     :    :                   

MG01523.1 	---
NCU08298.1	RKF
FG01969.1 	---
AN6211.1  	---
          	
```
